# Sleep quality and emergence delirium in children undergoing strabismus surgery: a comparison between preschool- and school-age patients

**DOI:** 10.1186/s12871-021-01507-2

**Published:** 2021-11-22

**Authors:** Wangseok Do, Hyo-Sung Kim, Seung Ha Kim, Hyunjong Kang, Dowon Lee, Jiseok Baik, Hyeon Jeong Lee, Jeong-Min Hong

**Affiliations:** 1 Biomedical Research Institute, Pusan National University Hospital, Busan, 49241 Republic of Korea; 2 Department of Anesthesia and Pain Medicine, School of Medicine, Pusan National University, 1-10, Ami-dong, Seo-gu, Busan, 49241 Republic of Korea

**Keywords:** Emergence delirium, Preoperative sleep quality, Pediatrics, Strabismus surgery

## Abstract

**Background:**

Emergence delirium (ED) is common in pediatric patients undergoing general anesthesia with sevoflurane. Preoperative sleep quality is associated with the risk factors for ED. However, research on the relationship between sleep quality and ED is limited. We aimed to investigate the relationship between ED and preoperative sleep quality in pediatric patients undergoing strabismus surgery.

**Methods:**

This clinical trial included pediatric patients aged 4–12 years who underwent elective strabismus surgery. The patients and their parents were questioned about the patients’ preoperative sleep quality using the Pittsburgh Sleep Quality Index (PSQI) questionnaire. For anesthesia induction, thiopental (5 mg/kg) and rocuronium (0.6 mg/kg) were used, and anesthesia was maintained with sevoflurane (minimum alveolar concentration, 1–1.5). After administration of a reversal drug, extubation was performed, and the patients were transferred to a post-anesthesia recovery unit. At 10 min after extubation, the degree of ED was measured using the pediatric anesthesia emergence delirium (PAED) and Watcha scale scores.

**Results:**

Of the 62 enrolled patients, three pediatric patients were excluded. The overall incidence of ED was 22%. A total of 59 patients were divided into the two groups. The ED group and the non-ED group comprised 13 and 46 patients. Age, height and weight were significantly lower in the ED group than in the non-ED group. Preoperative PSQI and Watcha scale score were significantly higher in the ED group than in the non-ED group. Multivariate analysis showed that age (adjusted OR [95% CI]: 0.490 [0.290–0.828], *p* = 0.008) and preoperative PSQI score (adjusted OR [95% CI]: 2.149[1.224–3.771], *p* = 0.008) was associated with ED. In sub-group analysis, PAED scale and Watcha scale scores showed a moderate correlation with preoperative sleep quality in preschool-age patients.

**Conclusion:**

In conclusion, the incidence of ED tended to be higher in younger age and poorer preoperative sleep quality in pediatric patients. In particular, the poorer sleep quality score was associated with higher incidence of ED in the preschool-age. Large-scale clinical studies and long-term follow-up studies on ED and sleep quality are required.

**Trial registration:**

This study was registered with Clinicaltrials.gov (NCT03332407) at November 5th 2017.

**Supplementary Information:**

The online version contains supplementary material available at 10.1186/s12871-021-01507-2.

## Background

Emergence delirium (ED) is defined as confusing mentality, irritability, inconsolable crying, and disorientation at the time of emergence from anesthesia [1]. To date, its physiological mechanism has not yet been fully elucidated. However, some possible risk factors for ED, such as preschool age, rapid emergence from inhalational anesthesia, presence of pain, stressful induction, noisy and unfriendly environment, duration of anesthesia, premedication, anxiety, anesthetic techniques, and patient’s personality, have been identified [1, 2]. According to previous reports, the incidence of ED varies from 0.25 to 95%, and it frequently occurs in pediatric patients. Fortunately, ED is a self-limiting phenomenon that lasts for approximately 5 to 15 min [3]. However, it has been reported to frequently cause catheter removal, attempts at self-extubation, and injuries to patients and staff [4]. In addition, children who experience ED during the post-anesthesia period can be exposed to a risk of falling from the bed or aggravation of wounds. Several studies have investigated the prevention of ED with drugs such as propofol, midazolam, ketamine and dexmedetomidine, given at the end of surgery. However, superiority over effective methods has not been defined [5–8].

Sleep is an essential factor in maintaining the physiological functions of the human body. Sleep is crucial for our daily living and related to the prognosis of patients treated under general anesthesia. If a person has a poor sleep quality preoperatively, it has been reported as a risk factor for severe postoperative pain [9–11]. Previous studies have reported that preoperative sleep quality is associated with postoperative delirium [12–15]. Postoperative pain, personality disorder, maladaptive behavior, anxiety, and postoperative delirium are associated with sleep quality and are also risk factors for the occurrence of ED [16–20]. However, no study has demonstrated the relationship between sleep quality and ED in pediatric patients. In particular, there have been no studies on differences in this relationship according to the age of children. We aimed to investigate the relationship between ED and preoperative sleep quality in pediatric patients undergoing strabismus surgery.

## Materials and methods

### Study design

The Institutional Review Board of Pusan National University Hospital, Busan, Korea, approved this study (1704-001-067). The investigators obtained informed consent from both patients and parents on the day of the pre-anesthetic evaluation visit.

The study protocol included patients of 4 to 12 years of age who underwent elective strabismus surgery. The components of exclusion criteria are children with disorders or taking medications known to affect blood pressure, sleep, breathing, and neuro-cognitive or behavioral functioning.

The investigators questioned the patients and their parents about their preoperative daily sleep quality using the Pittsburgh Sleep Quality Index (PSQI) questionnaire. The PSQI is a famously used measure of sleep-related problems and sleep quality over the past month [18, 21, 22]. The 19 items of the PSQI are grouped into seven components, scores of which reflect the frequency and severity of sleep problems, including subjective appreciation of sleep quality, sleep latency, sleep duration, habitual sleep efficiency, presence of sleep-disturbing factors, use of sleep medication, and daytime dysfunction. Each component is scored from 0 (no difficulty) to 3 (severe difficulty). All component scores are summed to provide an overall score ranging from 0 to 21, with a lower score indicating healthier sleep quality. A total score of 5 or greater is used to distinguish “poor” from “good” sleepers.

Before surgery, the patients were not pre-medicated. The parents accompanied their children in the operating room. Anesthesia was induced using thiopental (5 mg/kg) and rocuronium (0.6 mg/kg). Anesthesia was maintained with sevoflurane (minimum alveolar concentration, 1–1.5). Investigators performed routine monitoring for all patients, including pulse-oximetry, non-invasive blood pressure assessment, electrocardiography, and end-tidal CO_2_ monitoring. At the end of the surgery, reversal agents (glycopyrrolate [0.01 mg/kg and 0.25 mg/kg] and ketorolac [0.5–1.0 mg/kg]) were administered. The endotracheal tube was removed after recovery of cough and gag reflex and ability to grimace and perform purposeful movements. After extubation, the patients were transported to a post-anesthesia recovery unit (PACU).

As soon as the patients arrived at the PACU, the parents were called and asked to stand by the patients’ bedside. At 10 min after extubation, the degree of ED was measured using pediatric anesthesia emergence delirium (PAED) and Watcha scale scores. The PAED scale as shown in supplemental Table 1, has five items scored from 0 to 4 (with reverse scoring where applicable). The scores were summed to obtain a total score ranging from 0 to 20. A total score of 10 or greater was used to describe ED.

Meanwhile, the Watcha scale is a four-point scale, as shown in supplemental Table 2, and a score of 3 or 4 at any time indicates ED. The patient stayed in the PACU for 20 to 50 min, with vital sign monitoring including pulse-oximetry, non-invasive blood pressure assessment, and electrocardiography. The patients were discharged from the PACU based on a modified Aldrete score and the modified post-anesthetic discharge score.

### Statistical analysis

Statisticians performed all analyses using R (version 4.0.1) and they performed two-tailed tests at a significance level of 5%. The categorical variables were expressed as frequencies and percentages. The normal distribution of the data was tested using Kolmogorov–Smirnov test. The nonparametric data were presented as medians with interquartile ranges and parametric data as means ± standard deviations. The results of logistic regression analyses were presented as odds ratio (OR) or adjusted OR with 95% confidence interval (95% CI). The occurrence of ED was judged based on PAED scale score of 10 or greater, and patient data were analyzed by dividing them into ED and non-ED groups. Statisticians performed independent t-tests or Wilcoxon rank-sum tests on continuous variables. They also performed the chi-square test or Fisher’s test on categorical variables. Univariate logistic regression analysis was performed to evaluate the association between predictor variables and the occurrence of ED. The independent predictor variables include age, gender, height, weight, body mass index (BMI), anesthesia time, preoperative PSQI score, and ketorolac administered per kilogram. The candidate predictors enrolled in the multivariate logistic regression were those with *p*-values less than 0.05 in the univariate analysis. We performed subgroup analysis to elucidate the correlation between preoperative PSQI score and a PAED scale or a Watcha scale score. We classified the patients into two groups: preschool- and school-age groups. The preschool-age group included patients aged < 7 years, while the school-age group included patients aged ≥7 years. The correlation between the PAED scale scores and other factors was analyzed using a Spearman correlation analysis. Statistical significance was set at *p* < 0.05.

## Results

**Fig. 1:**
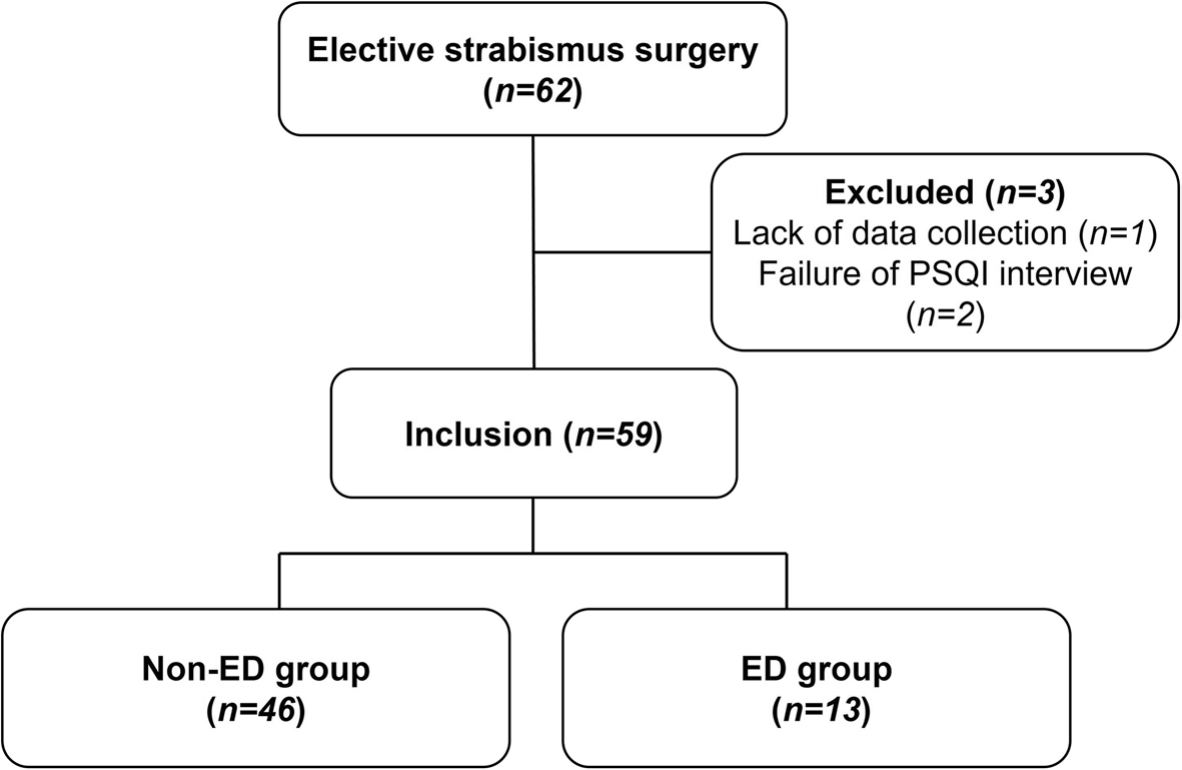
Study flow chart. PSQI Pittsburg Sleep Quality Index, ED Emergence delirium

Of the 62 enrolled patients, three pediatric patients were excluded because of a lack of data and failure to complete the PSQI interview. The study flowchart is displayed in Fig. 1.

The occurrence of ED was determined based on the PAED score, and the demographic data of the patients are presented in Table 1. The overall incidence of ED was 22%. A total of 59 patients were divided into the two groups. The ED group consisted 13 patients whereas, the non-ED group consisted only 46 patients. There were no significant differences between the two groups in gender, BMI, anesthesia time and amount of ketorolac administered per kilogram of body weight (*p* > 0.05). Age (*p* = 0.003), height (*p* = 0.008) and weight (*p* = 0.023) were significantly lower in the ED group than in the non-ED group. Preoperative PSQI (*p* = 0.028) and Watcha scale score (*p* < 0.001) were significantly higher in the ED group than in the non-ED group. The occurrence of ED was 11 of 29 (37.9%) in the preschool-age group and 2 of 30 (6.7%) in the school-age group. There was a statistically significant association between the occurrence of ED and age-group (*p* = 0.005).

**Table 1:** Patient demographics

	Overall (***n*** = 59)	Group		***P***-value
		ED (***n*** = 13)	Non-ED (***n*** = 46)	
**Age(y)**	6.71 ± 1.94	5.54 ± 1.33	7.04 ± 1.97	0.003
Pre-School age	29 (49.2)	11 (84.6)	18 (39.1)	0.005
School age	30 (50.8)	2 (15.4)	28 (60.9)	
**Gender**				
Female	31 (52.5)	6 (46.2)	25 (54.3)	0.835
Male	28 (47.5)	7 (53.8)	21 (45.7)	
**Height (cm)**	122.51 ± 14.00	114.94 ± 9.72	124.65 ± 14.36	0.008
**Weight (Kg)**	26.86 ± 10.17	22.86 ± 5.19	27.98 ± 10.97	0.023
**BMI (kg/m**^**2**^**)**	16.64 [15.51,18.37]	16.74 [16.24, 18.34]	16.49 [15.27, 18.38]	0.499
**Anesthesia time (min)**	45.00 [37.50,45.00]	45.00 [45.00,60.00]	45.00 [30.00,45.00]	0.327
**Pre-PSQI**	1.41 ± 1.65	2.38 ± 1.71	1.13 ± 1.54	0.028
**Ketorolac (mg/kg)**	0.63 ± 0.19	0.67 ± 0.16	0.62 ± 0.20	0.378
**Watcha scale**	2.54 ± 0.750	3.38 ± 0.650	2.30 ± 0.591	< 0.001

The incidence of emergence delirium defined on the basis of a PAED scale score of 10 or higher. Data are presented as numbers (%) or means *±* standard deviations or medians [interquartile ranges]

*BMI* Body mass index, *ED* Emergence delirium, *Pre-PSQI* Preoperative-Pittsburg Sleep Quality Index

Univariate analysis presented eight predictors which were expected to be associated with the occurrence of ED (Table 2). In univariate analysis, age (OR[95% CI]: 0.605 [0.399–0.919], *p* = 0.018), height (OR[95% CI]: 0.943 [0.894–0.995, *p* = 0.031]) and Preoperative PSQI score (OR[95% CI]: 1.554[1.069–2.259], *p* = 0.021) were statistically significant. Age, height and preoperative PSQI score were entered in the multivariate logistic regression analysis. The results of variable selection using the backward elimination method were age (adjusted OR [95% CI]: 0.490 [0.290–0.828], *p* = 0.008) and preoperative PSQI score (adjusted OR [95% CI]: 2.149 [1.224–3.771], *p* = 0.008). However, when the occurrence of ED was judged with a Watcha scale score of 3 or higher, preoperative PSQI score (adjusted OR [95% CI]: 1.077 [0.675–1.718], *p* = 0.756) showed no statistically significant results in multivariate analysis.

**Table 2:** Univariate and Multivariate analysis of variables related with emergence delirium

	Univariate analysis		Multivariate analysis	
	OR (95% CI)	*P*-value	Adjusted OR (95% CI)	*P*-value
Age	0.605 (0.399–0.919)	0.018	0.490 (0.290–0.828)	0.008
Gender	1.389 (0.404–4.776)	0.602		
Height	0.943 (0.894–0.995)	0.031	1.728 (0.753–3.967)	0.197
Weight	0.926 (0.842–1.017)	0.108		
BMI	0.965 (0.778–1.196)	0.744		
Anesthesia time	1.009 (0.977–1.042)	0.598		
Pre-PSQI	1.554 (1.069–2.259)	0.021	2.149 (1.224–3.771)	0.008
Ketolorac	3.994 (0.126–126.477)	0.432		

*BMI* Body mass index, *Pre-PSQI* Preoperative-Pittsburg Sleep Quality Index

The correlations of the PAED and Watcha scale scores with the preoperative PSQI were analyzed via a Spearman correlation analysis in the preschool- and school age group (Table 3). In the preschool-age group, there was a significant correlation between the PAED scale scores and preoperative PSQI (correlation coefficient = 0.377, *p* = 0.044). There was also a significant correlation between the Watcha scale score and preoperative PSQI (correlation coefficient = 0.376, *P* = 0.044). However, in the school-age group, there were no significant correlations between the preoperative PSQI and PAED (correlation coefficient = − 0.010, *p* = 0.957) or Watcha scale scores (correlation coefficient = 0.069, *p* = 0.712).

**Table 3:** Correlation coefficient between the preoperative PSQI and PAED and Watcha scale scores

		Preoperative PSQI	
		Preschool age (***n*** = 29)	School age (***n*** = 30)
PAED scale score	Correlation coefficient	0.377	- 0.010
	*P* value	0.044	0.957
Watcha scale score	Correlation coefficient	0.376	0.069
	*P* value	0.044	0.712

*PSQI* Pittsburg Sleep Quality Index, *PAED* Pediatric anesthesia emergence delirium

## Discussion

Our study demonstrated a correlation between preoperative sleep quality and postoperative ED in pediatric patients who underwent strabismus surgery. Our findings suggest that the poorer preoperative sleep quality and younger age were significantly correlated with the occurrence of ED. Also, in the preschool age group, higher preoperative PSQI scores were associated with higher PAED scale scores. That is, patients with poor preoperative sleep quality showed a higher incidence of ED in preschool age. Nevertheless, the etiology or pathogenesis of ED is unknown, so more research is needed to determine whether the poor preoperative sleep quality is simply accompanied by ED or the cause of ED.

Many factors affect sleep quality. These factors may be biological, psychosocial, or environmental. Biological factors include comorbid medical conditions (e.g., obesity, pain, and asthma), primary sleep disorders (e.g., obstructive sleep apnea and restless legs syndrome), and circadian rhythm changes in adolescents [23]. Psychosocial factors include depression, anxiety, personality disorder, substance abuse, family-related stressors (e.g., parental discord), and academic pressure [17, 20, 23]. Finally, environmental factors include multi-media usage, socioeconomic status, sleep habits, and sleep hygiene [23]. A previous adult-oriented study has shown that a worsening sleep quality was associated with preoperative anxiety [24]. Among the factors that affect sleep quality, obesity, anxiety, pain, personality disorder, and substance abuse are also risk factors for ED [1–4]. Considering these similarities, we hypothesized that there would be a relationship between ED and preoperative sleep quality.

In contrast to the preschool-age group, no association was found between preoperative sleep quality and PAED scale score in the school-age group. There are many factors regarding sleep quality and age can affect those factors itself. Family-related stress, academic pressure, and socioeconomic status have greater influence on sleep quality in the school-age group than in the preschool-age group. Therefore, in the school-age group, environmental factor may be dominant than in preschool-age group.

We enrolled patients between 4 and 12 years of age despite well known risk factor for ED age range is from 2 to 5 years [1]. The PSQI is based on a self-report of conditions and therefore requires proper communication and understanding of the patients. For this reason, children aged 2 to 3 years were ineligible for PSQI interview. Nevertheless, the results of this study were similar to those of previous studies [1, 3, 25, 26]. The preschool-age group scored higher on PAED and Watcha scale than the school-age group. Moreover, the preschool-age group had higher incidence of ED than the school-age group (37.9% vs. 6.7%). Although the participants’ ages in our study differed from those in previous studies, we could demonstrate noticeable age-related changes in the occurrence and severity of ED.

In our study, the overall incidence of ED was 22%, which was lower than that reported in previous studies [1–3, 26]. First, in our hospital, we allowed parents to stay with the patient before and after anesthesia. As a result, the patient’s anxiety level was expected to be relatively low. A child’s preoperative anxiety level is another well-known risk factor for ED [5, 27]. The factors related to increase in preoperative anxiety include separation anxiety, anxiety on operation anesthesia, and an unusual environment. Parental presence during anesthetic induction is controversial in reducing anxiety [28, 29]. Nevertheless, we allowed parents to come along before the induction of anesthesia. In addition, parents were again called to stay beside the patients in the PACU when the surgery was over. One of the parents was even called to stay beside the patient upon arrival at the PACU. Under such circumstances, the patients were more comfortable, less anxious, and cried less compared to when no parents are present in the PACU [30]. The scores of some items on the PAED scale, such as restlessness and inconsolability, might be lower in our study than in previous studies.

Second, adequate pain control with intravenous ketorolac appears to reduce the occurrence of ED. Postoperative pain is a risk factor for ED. We attempted to control postoperative pain with ketorolac (0.5–1.0 mg/kg, intravenous) in all patients (Table 1). Intravenous ketorolac injection is an effective pain control method in pediatric strabismus surgery [31, 32]. In our study, there was no difference in the dosage of ketorolac according to body weight between the two groups. Therefore, it is likely that postoperative pain did not significantly contribute to the differences between the two groups in our study.

Based on our findings, poor preoperative sleep was assumed to be a predictive factor for ED in pediatric patients. If there is a method to improve preoperative sleep quality, then it might be helpful to decrease the incidence of ED. Obstructive sleep apnea is known to cause sleep disruption and has already been reported in studies to be associated with postoperative delirium [12, 14]. Among the PSQI, items D “Cannot breathe comfortably” and E “Cough or snore loudly” of question 5 are questions related to obstructive sleep apnea. In our study, 50% of patients with a PSQI score of 5 or higher reported snoring in their daily sleep patterns. Several studies have improved sleep quality by correcting sleep hygiene and obstructive sleep apnea [23]. Therefore, further research is needed on the relationship between sleep quality improvement and the occurrence of ED when a modifiable factor of sleep quality is identified and corrected.

This study had some limitations. First, the most important limitation of our study is that a tool for assessing the quality of sleep in children has not been established. Although the PSQI questionnaire is an assessment tool used in previous studies on sleep quality in children, there seem to be some limitations in its application [18, 21, 22]. In the case of younger children, it was difficult to assess the PSQI due to communication problems; thus, children under the age of 4 were excluded from our study. This study obtained a relatively low PSQI (1.41 ± 1.65) score based on a commonly used cutoff value, which is not suitable for diagnosing sleep disturbance. This result may be because some of the questions in the PSQI questionnaire were not suitable for pediatric patients. PSQI is also not a good parameter for measuring short-term changes in sleep quality. This is because the PSQI questions have been used to assess sleep quality in the past month. The timing of PSQI interview was right after the surgery schedule confirmation. There could be preoperative anxiety which might influence the sleep quality before surgery that is not reflected in our PSQI score. To compensate for this aspect, other sleep quality assessment tools such as polysomnography might be added.

Second, preoperative and postoperative pain assessments were not performed. Pain is an important component of ED; therefore, it would have been better if it had been evaluated. However, intravenous ketorolac injection is effective for pain control in strabismus surgery and has been used routinely in all patients [31, 32]. As there was no difference in the amount of ketorolac used per kilogram of body weight, we assumed that the effect of pain was not significant. Third, in our study, we could not find a significant association between sleep quality and the occurrence of ED in the school-age group. However, since the incidence of ED (6.7%) was low in school-age group, it seems insufficient to determine the correlation between sleep quality and occurrence of ED. So further studies with more school age patients are needed.

## Conclusion

In conclusion, the incidence of ED tended to be higher in younger age and poorer preoperative sleep quality in pediatric patients. The poorer sleep quality score was associated with higher incidence of ED in the preschool-age. In consideration of the limitations of our study, further studies are required to investigate the correlation between preoperative sleep quality and postoperative ED.

## Supplementary Information

**Additional file 1 **: **Supplementary Table 1.** Pediatric anesthesia emergence delirium scale. **Supplementary Table 2.** Watcha scale.

## Data Availability

Data are available from the corresponding author on reasonable request.

## Abbreviations

BMI: Body mass index
CI: Confidence interval
ED: Emergence delirium
OR: Odds ratio
PSQI: Pittsburg sleep quality index
PAED: Pediatric anesthesia emergence delirium
PACU: Post-anesthesia recovery unit
