# Supplementary material for: Sleep quality and emergence delirium in children undergoing strabismus surgery: a comparison between preschool- and school-age patients
Source: BMC Anesthesiol. 2021 Nov 22;21:290. doi: 10.1186/s12871-021-01507-2 (PMC8607612; doi:10.1186/s12871-021-01507-2)
Supplement: Supplementary file 1 — Additional file 1 : Supplementary Table 1. Pediatric anesthesia emergence delirium scale. Supplementary Table 2. Watcha scale. [file 12871_2021_1507_MOESM1_ESM.docx]

**Sleep quality and emergence delirium in children undergoing strabismus surgery: a comparison between preschool- and school-age patients**

Wangseok Do^2^, Hyo-Sung Kim^2^, Seung ha Kim^2^, Hyunjong Kang^2^, Dowon Lee^1,2^, Jiseok Baik^1,2^, Hyeon-Jeong Lee^1,2^, Jeong-Min Hong^1,2^

^1^ Department of Anesthesia and Pain Medicine, School of Medicine, Pusan National University, Busan 49241, Republic of Korea

^2^ Biomedical Research Institute, Pusan National University Hospital, Busan 49241, Republic of Korea

**Corresponding author: Jeong-Min Hong, MD, PhD,** Department of Anesthesia and Pain Medicine, School of Medicine, Pusan National University, 1-10, Ami-dong, Seo-gu, Busan 49241, Republic of Korea. [ccarrot@pusan.ac.kr](mailto:ccarrot@pusan.ac.kr)

**Supplementary Tables:**

**Supplementary Table 1: Pediatric anesthesia emergence delirium scale.**

| **Pediatric anesthesia emergence delirium scale** | | | | | |
| --- | --- | --- | --- | --- | --- |
| **Scale** | **Not at all** | **Just a little** | **Quite a bit** | **Very much** | **Extremely** |
| The child makes eye contact with the care giver | 4 | 3 | 2 | 1 | 0 |
| The child’s actions are purposeful | 4 | 3 | 2 | 1 | 0 |
| The child is aware of his/her surroundings | 4 | 3 | 2 | 1 | 0 |
| The child is restless | 0 | 1 | 2 | 3 | 4 |
| The child is inconsolable | 0 | 1 | 2 | 3 | 4 |

The scores were summed to obtain a total score ranging from 0 to 20. A total score of 10 or greater was used to describe emergence delirium

**Supplementary Table 2 : Watcha scale.**

| **Watcha Scale** | |
| --- | --- |
| The child is calm | 1 |
| The child is crying, but can be consoled | 2 |
| The child is crying and cannot be consoled | 3 |
| The child is agitated and thrashing around | 4 |

Watcha scale is a four-point scale, and a score of 3 or 4 at any time indicates emergence delirium
